# Clinical spectrum, treatment and outcomes of the m.10197G>A mutation in MT-*ND3*: a case report, systematic review and meta-analysis

**DOI:** 10.1186/s13023-025-03588-5

**Published:** 2025-02-08

**Authors:** YuZhi Shi, Bin Chen, SongTao Niu, XinGao Wang, ZaiQiang Zhang

**Affiliations:** Department of Neurology, Beijing Tiantan Hospital, Capital Medical University, No. 119, South 4th Ring Road West, Fengtai District, Beijing, 100070 China

**Keywords:** *ND3*, Leigh syndrome, Leber hereditary optic neuropathy, Dystonia, Mitochondrial disease

## Abstract

**Background:**

A correlation between various sites or types of mutations in mitochondrial DNA *ND3* and the development of a specific mitochondrial disease or phenotype has yet to be fully established.

**Methods:**

This study reports a rare case of adult-onset Leigh syndrome (LS) and Leber hereditary optic neuropathy and dystonia (LDYT) overlap syndrome caused by the m.10197G>A mutation in *ND3*. A review of the literature was conducted to investigate the clinical spectrum, treatment and outcome resulting from the m.10197G>A mutation. Phenotypes associated with the m.10197G>A mutation were classified into three categories: LS/LS+ (LS-involved overlap syndrome), Leber hereditary optic neuropathy (LHON)/LHON+ (LHON-involved overlap syndrome) and other mitochondrial encephalopathies or presentations.

**Results:**

A total of 84 participants (78 patients and 6 asymptomatic carriers) with the m.10197G>A mutation retrieved from 33 articles and the patient whose case we reported were included in the review and meta-analysis. Among all the participants, 55.3% (47/85) and 28.2% (24/85) presented with LS/LS+ and LHON/LHON+, respectively. The median age at onset for LS/LS+ was significantly younger than that for LHON/LHON+ [median, (Q1–Q3), 3.0 (0.58–9.5) vs. 13.5 (5.75–41.75), *P* = 0.001]. A negative linear correlation was observed between mutation load and age of onset in patients who presented with LS/LS+ (R^2^ = 0.592, *P* < 0.001), with the age of onset ranging from infancy to adulthood. Patients with an older age at onset [OR (95% CI), 1.46 (1.12–1.91), *P* = 0.005] or higher mutation loads [OR (95% CI), 1.14 (1.03–1.26), *P* = 0.011] were more likely to present with LHON/LHON+ than with LS/LS+. A total of 17 patients were documented as having received a combination of mitochondrial cofactor treatments. Compared with patients with LHON/LHON+, patients with LS/LS+ exhibited an exceedingly high probability of a stable or worsen outcome (93.8% vs. 33.3%, *P* = 0.006).

**Conclusions:**

LS/LS+ and LHON/LHON+ are the predominant presentations of the m.10197G>A mutation. An older age at onset and greater mutation load increases the probability of an LHON/LHON+ presentation. Patients presenting with LS/LS+ have an exceedingly high possibility of an unfavorable outcome. The identification of factors and outcomes associated with phenotypes in patients with the m.10197G>A mutation facilitates the provision of improved prognostic counseling for patients and their family members who are carriers of this mutation.

**Supplementary Information:**

The online version contains supplementary material available at 10.1186/s13023-025-03588-5.

## Introduction

Mitochondrial complex I is one of the major respiratory complexes, and plays a central role in oxidative energy metabolism, NADH oxidation, quinone reduction, and proton pumping across the membrane [1]. Isolated complex I deficiency is the most common genetic defect in childhood mitochondrial respiratory chain disorders, accounting for up to 30% of all cases of oxidative phosphorylation system disorders [2]. ND3 is one of the seven subunits of complex I; it is located in the hydrophobic arm of the membrane and is encoded by mitochondrial DNA (mtDNA).

The m.10197G>A variant results in the change of a hydrophobic alanine residue into a hydrophilic threonine (A47T) in a highly conserved domain of *ND3* and is a causative mutation. This variant was reported as pathogenetic mutation for the first time in a patient who presenting with Leigh syndrome (LS)-like mitochondrial encephalopathy in 2004 [3]. It was subsequently confirmed as a causative mutation of LS and dystonia in Korean and French patients [4, 5]. In 2009, Wang et al. reported 18 patients in a Chinese pedigree harboring the m.10197G>A mutation who presented with Leber hereditary optic neuropathy (LHON) or Leber hereditary optic neuropathy and dystonia (LDYT) [6]. Leng et al. confirmed that the m.10197G>A variant is a pathogenic mutation for mitochondrial encephalomyopathy with lactate acidosis and stroke-like episodes (MELAS)/LS overlap syndrome [7]. The m.10197G>A mutation is now considered a less frequent causative variant for LS or late-onset LS (age of onset > 2 years) [8–12], dystonia, MELAS, and LHON, and some mitochondrial overlap syndromes, such as LDYT, MELAS/LS, and LS/dystonia [13–15].

A correlation between the different sites or types of mutations in *ND3* and the development of a specific mitochondrial disease or phenotype has not been well established. Among all the reported clinical manifestations related to mitochondrial disease caused by the 10197G>A variant in *ND3*, we noticed that LS and LHON may be the main presentations in these patients. We report a case of a patient from a Chinese family harboring the m.10197G>A variant who presented with adult-onset LS/LDYT overlap syndrome, which spans the two major phenotypes associated with the m.10197G>A variant. In addition, we conducted a literature review of previously reported cases and analyzed the phenotypes, treatment and outcomes of patients to identify the clinical phenotypes of the m.10197G>A variant and associated factors.

## Methods

### Case report

Comprehensive medical and family history information was obtained. The results of the physical examination at admission were recorded.

#### Neuroimaging and laboratory examination

Brain magnetic resonance (MR) and spinal cord MR were performed repeatedly to diagnose and evaluate lesion progression during the proband’s disease process. The MR sequences included T1- and T2-weighted, fluid-attenuated inversion recovery (Flair) and susceptibility-weighted imaging (SWI) sequences. Brain MR spectroscopy (MRS) was used to detect the metabolic and biochemical changes in the brain lesions compared with healthy brain tissue.

Blood lactic acid levels were tested in the resting state.

#### Ophthalmologic examination

The patient underwent visual acuity measurement, indirect ophthalmoscopy, visual field testing, and optical coherence tomography (OCT) to assess the thickness of the retinal fiber layer (RNFL) and ganglion cell complex (GCC).

#### Function evaluation

The function of the proband was evaluated using Section I of the Newcastle Mitochondrial Disease Adult Scale (NMDAS) on admission and at the 1-year follow up. The NMDAS is a validated method to measure and monitor disease manifestations and clinical features in patients with mitochondrial disease [16].

#### Genetic analysis

EDTA-treated peripheral blood samples were obtained from the proband and his mother. Genomic DNA and mitochondrial DNA were extracted using the Blood Genome Column Medium Extraction Kit (Kangweishiji, China) and the Mitochondrial DNA Extraction Kit (Kangweishiji, China), respectively.

The protein-coding exome was enriched by the xGen Exome Research Panel v2.0 (IDT, Iowa, USA) and sequenced by the DNBSEQ-T7 (PE150) genetic sequencer (BGI, China). The next generation sequencing (NGS) data were aligned to the reference sequence Ensemble GRCh37/hg19 genome. The identification of single-nucleotide polymorphisms (SNPs) and insertion-deletion mutations (Indels) were performed using the GATK, Samtools, and Pindel software packages. The annotation and pathogenicity prediction were developed by the Chigene online system (www.chigene.org), which is based on American College of Medical Genetics (ACMG) practice guidelines, over 30 public databases, and published literature.

Mitochondrial DNA libraries were constructed using mitochondrial full-length PCR products as DNA templates and the VAHTS Universal Plus DNA Library Prep Kit for Illumina reagents, following the protocol for full-length mitochondrial genome amplification. The mitochondrial DNA was subjected to next-generation sequencing using Novaseq6000 sequencing system (Illumina, USA). The NGS data were aligned to the reference sequence Ensemble GRCh37/hg19 genome and the mitochondrial genome NC_012920 BWA after undergoing quality filtering with FASTP. The depth and quality of the reads were adjusted to screen reliable variants. Variants were mapped to reference mutations to find matches in the MITOMAP human mitochondrial genome database. The GATK variant detection tool was employed to identify variant loci and determine the mutation load based on their allele frequencies. The loci of the resultant variants, which were included in the MITOMAP database as “Confirmed pathogenic” and “Reported” were annotated using the VEP, and the frequency information was filtered according to the sample type. The structural variants were analyzed using PICARD, LUMPY and SVTYPER to obtain results on large segment deletion. The mutation load of the variant was calculated by the ratio of the number of reads of the variant to the total number of reads at this locus based on the NGS data.

### Literature review

**Fig. 1:**
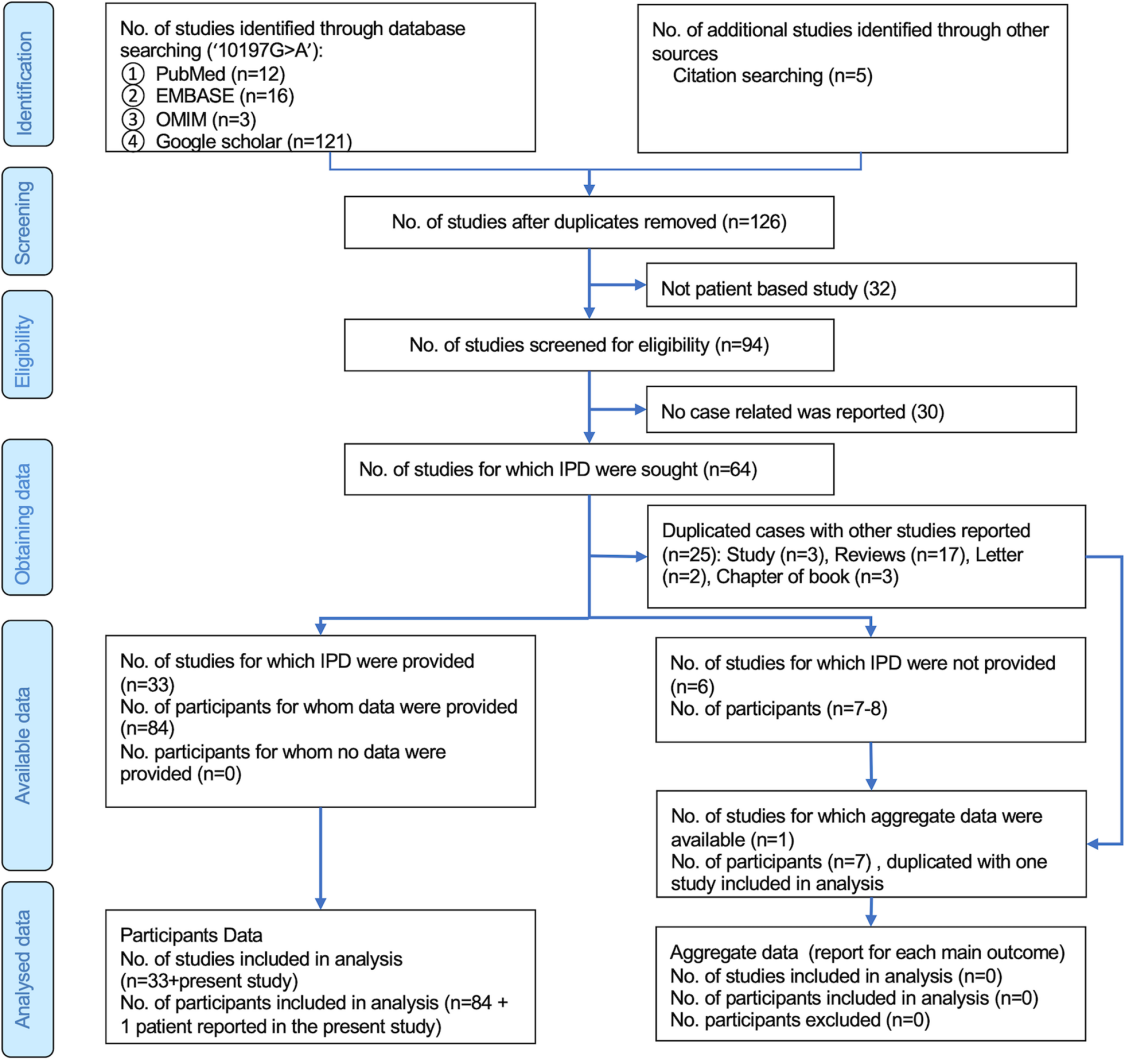
The PRISMA-IPD flow diagram

A literature review was conducted in accordance with the Preferred Reporting Items for Systematic Reviews and Meta-Analyses for Individual Patient Data (PRISMA-IPD) systematic review guidelines [17, 18]. A systematic search for the term “10197G>A” in all fields yielded 126 results from January 2004 to May 2024 in the PubMed, EMBASE, OMIM and Google Scholar databases without language restrictions [19, 20]. The retrieved articles were screened for reports, studies, or reviews associated with the phenotypes caused by the m.10197G>A variant. A total of 94 articles met the inclusion criteria and were further screened for an explicit description of the phenotypes. Ultimately, 33 articles, comprising 84 participants and the patient described in the present study, were included in the meta-analysis (Supplementary Table 1). Aggregate data from seven patients with the m.10197G>A mutation were identified in a study by Shi et al. [8], and these data were duplicated in another study [21] included in the review. Due to this duplication, aggregate data were not included in the analysis (Fig. 1). The information extracted from each article included the year of publication, age at onset and presentation, gender, heteroplasmy level, phenotypes, treatments and outcomes.

The phenotypes associated with the m.10197G>A mutation were classified into three groups: LS/LS+ (LS-involved overlap syndrome, including dystonia/LS, MELAS/LS, and LS/arterial malformation), LHON/LHON+ (LHON-involved overlap syndrome, including LDYT) and other forms of mitochondrial encephalopathy or presentation. Two senior neurologists independently reviewed the clinical data for each participants to determining the most appropriate classification of the observed phenotypes. A third senior neurologist was consulted for a decision in cases with discrepancy in classification. Given that LS was the initial and primary presentation of the proband, which is the case we reported in the present study, the proband was classified into the LS/LS+ group.

The treatments that targeted mitochondrial impairment and the outcomes at follow-up were recorded. The outcomes were classified into two categories: those who demonstrated recovery or relief of neurological function impairment at follow-up were classified as clinical improvement, and those who exhibited stability, deterioration of neurological function impairment, or death at follow-up were classified as stable or clinical worsening.

#### Statistical analysis

The statistical analysis was performed using SPSS 22.0 software (IBM, USA). The numerical variables are presented as median and quartiles (Q1, Q3). Categorical variables are presented as numbers and percentages. A one-sample Chi-square test was used to compare the gender and categories of phenotype differences among the participants. A nonparametric test was employed to compare the age of onset and heteroplasmy level between the LS/LS+ group and the LHON/LHON+ group. The relationship between the age of onset and mutation load was investigated through the use of linear regression. A logistic regression analysis was conducted to determine the factors associated with the presentation of LS/LS+ and LHON/LHON+ using the LS/LS+ group as the reference.

A two-tailed probability value of *P* < 0.05 was considered statistically significant. The Chi-square test used the Bonferroni adjustment in the pairwise comparison of the distribution of LS/LS+ and LHON/LHON+ in different mutation forms (heteroplasmy *vs.* homoplasmy). *P* < 0.0167 was considered statistically significant.

## Results

### Case report

#### Medical history and neurological examination

A male patient in his early 20 s was referred to the neurology department with a chief complaint of slurred speech and movement disorders. No abnormality in physical or psychomotor development was observed by his parents during his prenatal or in childhood periods. His mother began to develop vision disturbance, epilepsy, and abnormal gait in her second decade. A pedigree chart of the family is presented in Supplementary Fig. 1A.

The patient began to display a gait abnormality and left upper limb stiffness before 20 years of age, followed by right upper limb rigidity and visual disturbance 1 year later. His gait disturbance gradually worsened to the extent that he was unable to walk without assistance. Three months prior to the patient’s visit, he began to exhibit slurred speech and spontaneous spasm in his right biceps. The spasm occurred 4 ~ 5 times a day and lasted for seconds at a time.

The neurological examination revealed poor visual acuity (OD 0.15, OS 0.3), severe dysarthria, upper and lower limbs hyper myotonia, and lower limbs hyperreflexia. His right upper limb was unable to be flexed.

#### Laboratory and neuroimaging findings

**Fig. 2:**
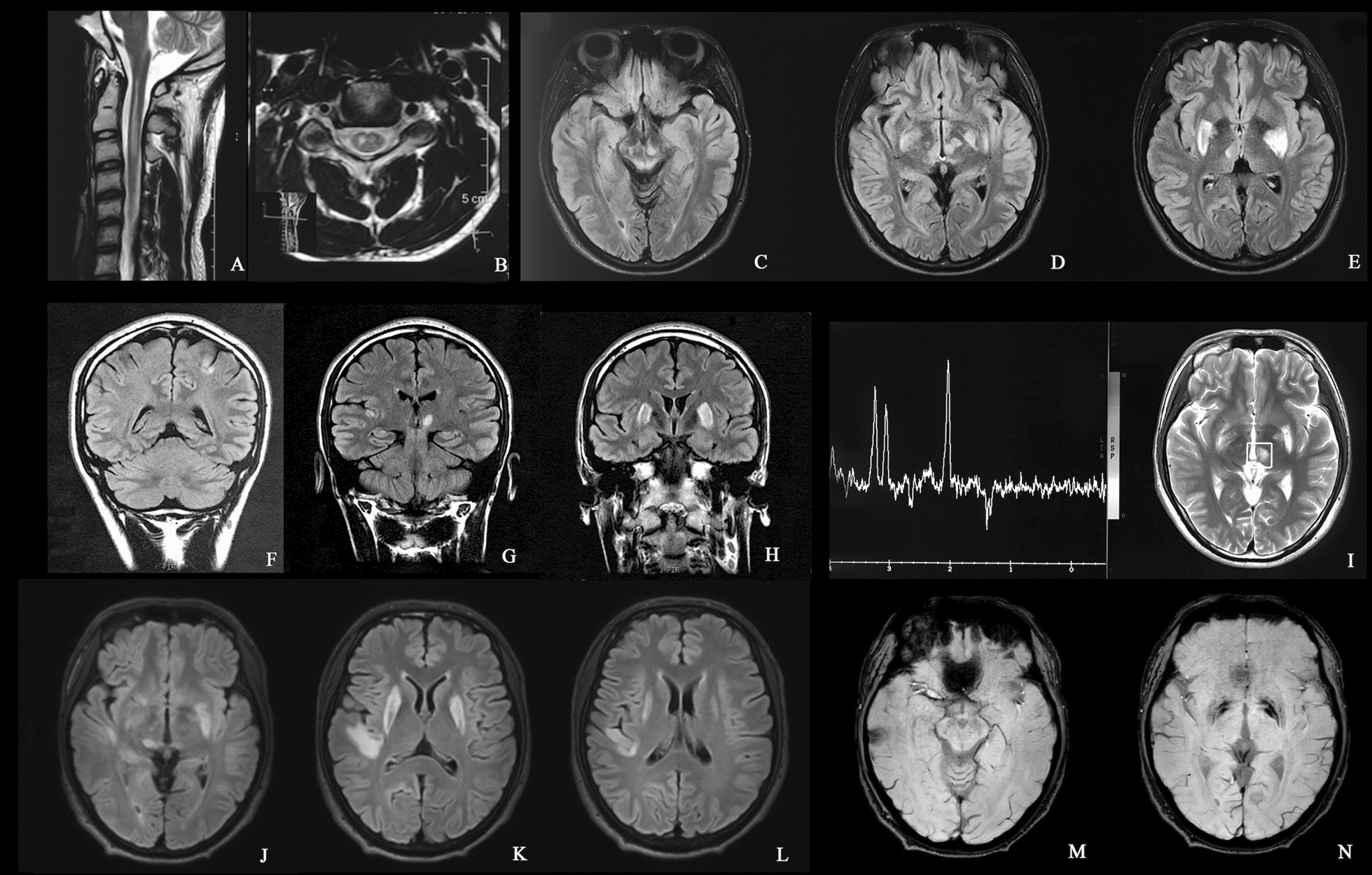
Neuroimaging of the patient. In the year of the onset of neurological disorders, neuroimaging examination revealed lesions in the cervical spinal cord, bilateral lenticular nuclei, thalamus, and mesencephalon (**A**–**E**). New lesions were detected in the left parietal cortex and thalamus 1 year after onset (**F**–**H**). Magnetic resonance spectroscopy (MRS) showed inverted lactate peaks with normal *N*-acetyl aspartate in the lesion of the left thalamus (**I**). Brain MR was conducted for his new symptoms, and new lesions in the right thalamus and right temporal cortex (**J**–**L**) were observed. Hemosiderosis was observed in the midbrain lesions and bilateral lenticular nuclei (**M**, **N**)

The blood lactate level was 3.28 mmol/L (normal value 0.7–2.1 mmol/L) in the resting state. Magnetic resonance imaging (MRI) of the cervical spinal cord and brain performed at the onset of neurological disorders showed abnormally high-signal lesions in the upper cervical spinal cord (Fig. 2A, B), mesencephalic gray matter, periaqueductal region, and bilateral lenticular nuclei (Fig. 2C–E). Involvement of the bilateral thalamus and the parietal and temporal cortex was identified in the subsequent 2 years (Fig. 2F–L). MRS demonstrated inverted lactate peaks in an acute lesion in the left thalamus (Fig. 2I). Hemosiderosis deposition was observed in lesions located in the mesencephalon and bilateral lenticular nuclei (Fig. 2M, N).

#### Ophthalmologic examination

**Fig. 3:**
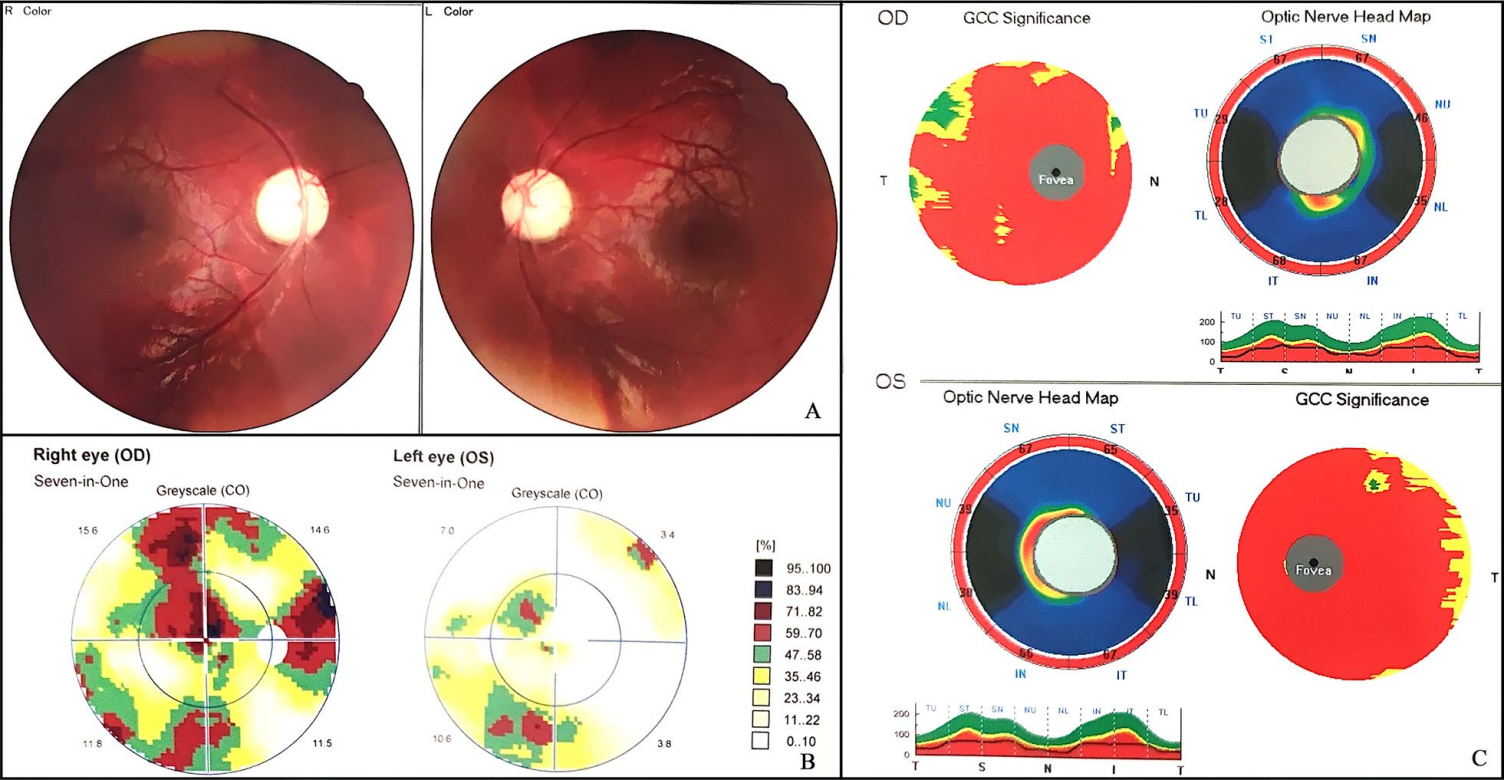
Ophthalmologic assessment of the patient. The optic disks were pale, and the retina was flat according to fundus examination (**A**). Vision field examination showed irregular visual field defects, which were especially severe in the right eye (**B**). Optical coherence tomography displayed decreased retinal nerve fiber layer thickness and a loss of the macular retinal ganglion cell complex (**C**)

The ophthalmologic assessment revealed pale optic disks and irregular defects in the visual field. The RNFL thickness decreased, with an average of 50.72 μm in the right eye (OD) and 51.00 μm in the left eye (OS). GCC exhibit a loss of thickness with average of 60.01 μm in the right eye (OD) and 55.77 μm in the left eye (OS) (Fig. 3).

#### Genetic sequencing

mtDNA sequencing revealed a variant in *ND3,* m.10197G>A, with heteroplasmy levels of 58.12% [322 / (322 + 232)] and 16.98% [54 / (54 + 264)] in blood samples from the proband and his mother, respectively, (Supplementary Fig. 1B), and 77.1% in muscle from the proband, according to NGS data of mtDNA. WES did not identify any causative variant associated with the clinical presentations.

#### Treatment and prognosis

The patient was treated with idebenone (90 mg/day) and ubidecarenone (30 mg/day). At the 1-year follow-up, the functional impairment of the proband neither improved nor deteriorated, according to the score of Section I of the NMDAS (27 *vs.* 26).

### Literature review

A total of 84 participants (78 patients and 6 asymptomatic carriers) harboring the m.10197G>A variant from the 33 articles and the patient whose case we reported above were included in the review and meta-analysis (Supplementary Table 1). Thirty-nine participants (39/85, 45.8%) from 13 families reported a positive family history. Following the exclusion of 19 participants for whom sex was not specified in the literature, the percentage of males was found to be higher than that of females (44.7% vs. 32.9%).

#### Phenotypes and classification

**Fig. 4:**
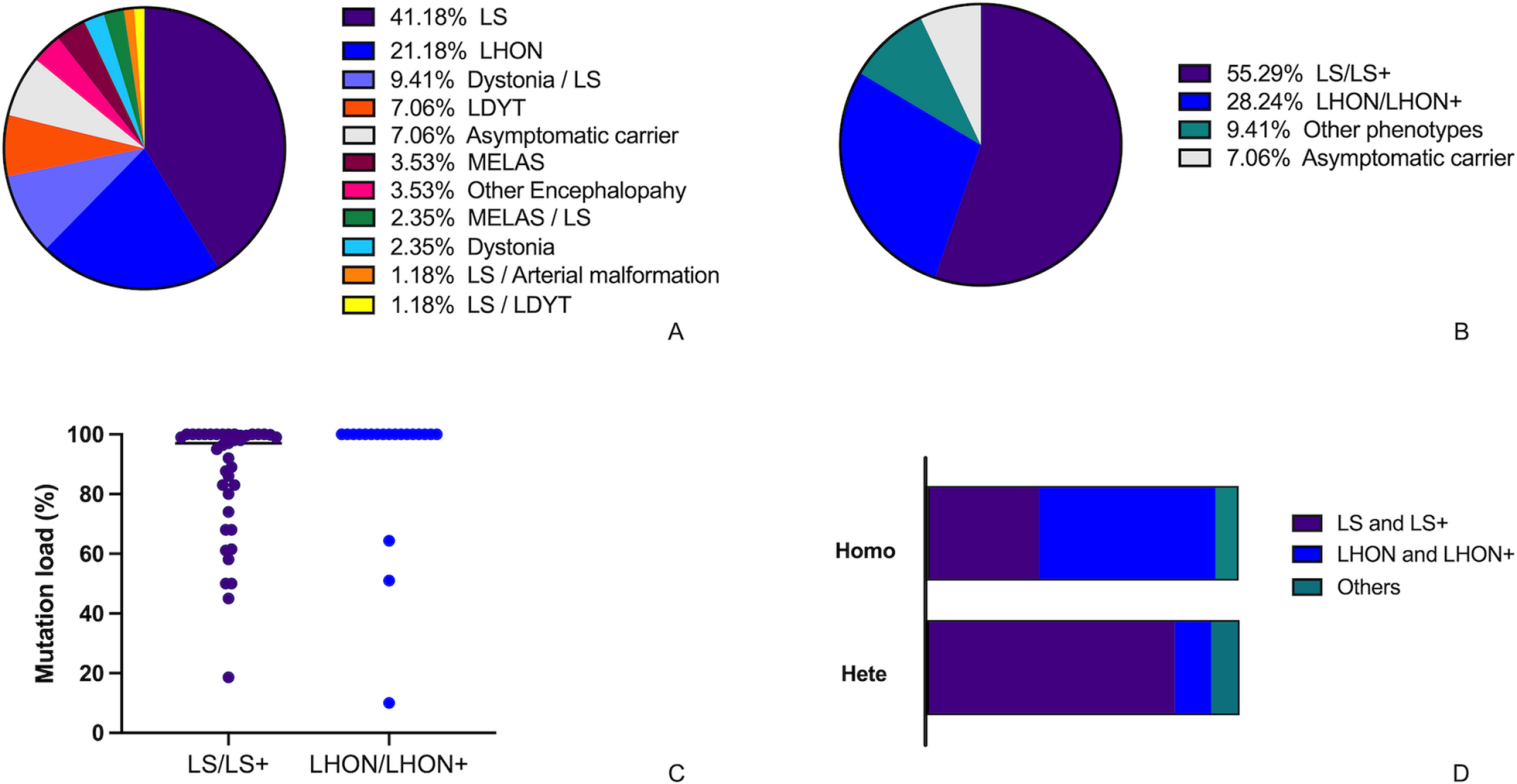
Clinical features of the m.10197G>A variant. The phenotype spectrum of patients with the m.10197G>A variant (**A**). The percentage of phenotype categories of clinical presentations in patients with the m.10197G>A variant (**B**). The mutation loads of the m.10197G>A variant in the LHON/LHON+ group and the LS/LS+ group (*P* < 0.001) (**C**). Phenotype categories in patients carrying the homoplasmic mutations and in patients carrying heteroplasmic mutations (*P* < 0.001) (**D**)

The two most prevalent phenotypes observed in all participants harboring the m.10197G>A variant were LS (n = 35, 41.2%) and LHON (n = 18, 21.2%), followed by dystonia/LS overlap syndrome (n = 8, 9.4%) and LDYT (n = 6, 7.1%) (Fig. 4A). LS/LS+ (n = 47) and LHON/LHON+ (n = 24) constituted 55.3% and 28.2% of all participants, respectively (Fig. 4B).

#### Age at onset

The age at onset of neurological or ophthalmic disorders in patients ranged from 0 to 58 years. Following the exclusion of 22 patients for whom age at onset data were unavailable, the median age of onset was 5.0 (Q1-Q3, 0.95–14.0) years in the remaining 57 patients. The median age of onset for LS/LS+ patients was significantly younger than that for LHON/LHON+ patients [median, (Q1-Q3), 3.0 (0.58–9.5) vs. 13.5 (5.75–41.75), *P* = 0.001].

Adult-onset neurological or ophthalmic disorders were reported in 11 patients. The phenotypes of these adult-onset patients included LS (n = 3), LHON (n = 7), and LS/LDYT overlap syndrome (n = 1). In addition to the seven patients with adult-onset LHON mentioned above, some patients with LHON began to develop vision disturbance after 18 years of age within the family, as reported by Wang et al. However, these cases were not included in the analysis of age at onset, as the age at onset was not reported individually. The number of adult-onset patients in the aforementioned pedigree was not available for analysis. The LHON phenotype was more prevalent than the LS phenotype in these adult-onset patients.

#### Mutation load

Mutation loads were individually reported in 70 (84.3%) participants, as detected using blood, urine, or tissue samples (skeletal muscle, fibroblasts, liver, buccal mucosa, etc.), and one patient reported a heteroplasmy mutation without a specific mutation load (Supplementary Table 1). The heteroplasmy levels of m.10197G>A were recorded in the review for analysis primarily according to the mutation levels in leukocytes (n = 56) and secondly according to the mutation levels in skeletal muscle for which blood data were not available (n = 12). The sample type was not specified in 3 patients.

The mutation load varied from 10.0% to 100% across all reported participants. The heteroplasmy levels in patients who presented with LS/LS+ and LHON/LHON+ ranged 18.6 ~ 100% and 10 ~ 100%, respectively. The mutation load in the LHON/LHON+ group was significantly higher than that in the LS/LS+ group [median (Q1, Q3), 100% (100–100%) vs. 97.0% (74.0–100%), *P* = 0.002] (Fig. 4C).

A total of 33 (39.7%) participants (2 asymptomatic participants and 31 patients) were found to harbor a m.10197G>A homoplasmic mutation. The proportion of patients who carried a homoplasmic mutation in the LHON/LHON+ group was much higher than in the LS/LS+ group (81.0% vs. 30.8%, *P* < 0.001). Significantly differences were observed between the categories of phenotypes in patients carrying the homoplasmic mutation and those carrying the heteroplasmic mutation (*P* < 0.001) (Fig. 4D). In patients with homoplasmic mutations, the LHON/LHON+ phenotype was more prevalent than the LS/LS+ phenotype (54.8% vs. 38.7%), whereas the LS/LS+ phenotype was significantly more common in those with heteroplasmic mutations (79.4% vs. 11.8%) (Fig. 4D).

#### The relationship between age of onset and mutation load

**Fig. 5:**
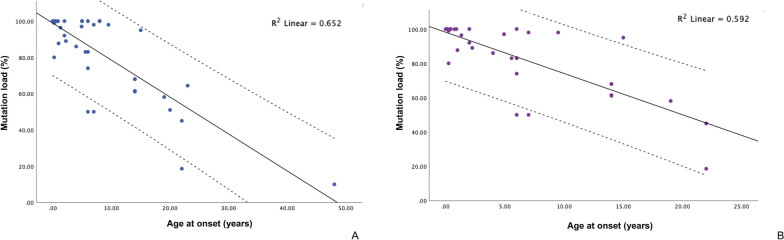
Correlations between mutation load and age of onset in patients regardless of phenotype (n = 42) (**A**) and in the LS/LS+ group (n = 33) (**B**)

A negative linear correlation was identified between the mutation load and the age of onset (R^2^ = 0.652, *P* < 0.001) in patients, regardless of phenotype, based on data from 42 patients (Fig. 5A). The majority of patients included in the linear regression model presented with the LS/LS+ phenotype (n = 32). A similar inverse correlation was identified between the age of onset and mutation load in the LS/LS+ group (R^2^ = 0.592, *P* < 0.001) (Fig. 5B). However, the aforementioned relationship could not be analyzed in the LHON/LHON group due to the absence of data on the age of onset or mutation load in 62.5% of cases. The data of m.10197G>A heteroplasmy in muscle samples were available for 21 patients, all of whom were from the LS/LS+ group. The relationship between the heteroplasmy level in muscles and the age of onset was investigated using linear regression analysis. The analysis included 17 patients, but no significant linear correlation was identified.

#### Variables associated with the phenotype categories

The variables of age at onset, mutation load, and sex were entered into the regression model. Compared to the presentation of LS/LS+, patients who had older age at onset [OR (95% CI), 1.46 (1.12–1.91), *P* = 0.005] or harbored higher mutation loads [OR (95% CI), 1.14 (1.03–1.26), *P* = 0.011] were more likely to present with the LHON/LHON+.

#### Incomplete penetrance

Among the 13 pedigrees that were found to harbor the m.10197 G>A mutation, 6 participants from 4 pedigrees (3 LS/LS+ pedigrees and 1 LHON/LHON+ pedigree) were identified as asymptomatic carriers. The age at presentation was reported in 2 asymptomatic carriers from two distinct pedigrees, one of which was LHON+ and the other was LS+. In both cases, the age at presentation was older than the oldest age at onset of ophthalmic or neurological disorders in their respective pedigrees (34 years vs. 30 years, 7 years vs. 6 years, respectively). The mutational load of the m.10197G>A mutation in these asymptomatic carriers ranged from 37 to 100% (Supplementary Table 1).

#### Treatment and outcomes

A total of 17 patients were reported to have received cocktail mitochondrial cofactor treatments, comprising idebenone, coenzyme Q10, and vitamins C, B and E. One female LHON patient, aged 48 years, was treated with hormone replacement therapy (etonogestrel 0.12 mg and ethinyl estradiol 0.015 mg) in addition to idebenone and vitamin C [22].

**Fig. 6:**
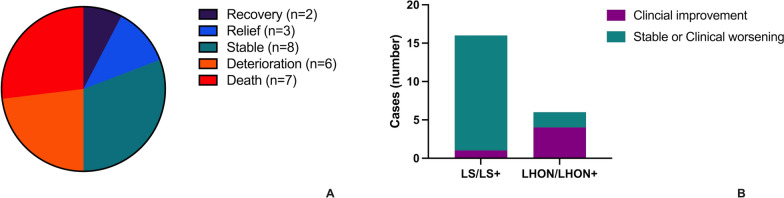
Proportion of outcomes in patients with the m.10197G>A mutation (**A**). The number of patients exhibiting clinical improvement and stable or clinical worsening in the LS/LS+ group and in the LHON/LHON+ group (**B**)

A total of 26 patients (21 LS/LS+ patients and 6 LHON/LHON+ patients) had reported outcomes, with only 19.2% (n = 5) of demonstrating clinical improvement (Fig. 6A). The only two patients who achieved complete recovery were diagnosed with LHON and experienced reversal of vision loss after a period of 8 months or 1 year of treatment [22, 23]. A 16-year-old female patient with a mild clinical manifestation of LS was treated with coenzyme Q10 for 3 months, resulting in improvements in exercise tolerance and anorexia, as well as weight gain. However, the neurological examination revealed no significant alterations [24]. The remaining two patients, who exhibited improved neurological impairment were diagnosed with LHON. The proportion of stable or worsening outcomes in the LS/LS+ group was markedly greater than that in the LHON/LHON+ group (93.8% *vs.* 33.3%, *P* = 0.006) (Fig. 6B).

## Discussion

We reported a rare case of adult-onset LS/LDYT overlap syndrome, spanning the two main phenotypes of patients harboring the m.10197G>A variant: LS/LS+ and LHON/LHON+ and conducted a literature review and meta-analysis to identify the clinical spectrum, treatment and outcome of the m.10197G>A mutation and factors associated with phenotype categories.

LS is the most common pediatric mitochondrial disease. The median age of onset is 1.1 years (interquartile range, 2.1 years), and one third of cases present with symptoms after 2 years of age and are categorized as late-onset LS [21]. Late-onset LS often exhibits atypical clinical features [25]. The m.10197G>A mutation is a predominantly causative mutations for late-onset LS [21, 25]. Our findings revealed that approximately half of the patients in the LS/LS+ group were classified as late-onset LS, a proportion notably higher than that in general LS patients (34%) [21].

The literature review identified three adult-onset LS cases [26–28], along with the LS/LDYT overlap syndrome case we reported. The proband exhibited syndromes spanning two major phenotypes associated with the m.10197G>A variant: the LS+ spectrum and the LHON+ spectrum. Digenic LS has been observed in cases with the m.11778A>G LHON variant and heterozygous variants in *NDUFS2, NDUFS7*, and *NDUFS8* [29]. Whole exome and mitochondrial DNA sequencing revealed no other suspicious variants that could explain the LS/LDYT overlap spectrum in the proband. Advances in digenic inheritance research in mitochondrial diseases may facilitate a more comprehensive understanding of the etiology of the LS/LDYT overlap spectrum [30]. On the other hand, this is the first report of dystonia caused by the m.10197G>A mutation in an adult-onset case.

In our review and meta-analysis, the median age of onset in the LHON/LHON+ group caused by the m.10197G>A mutation was 13.5 years, which was significantly younger than the previously reported mean age of LHON (27.9 years) [31]. Some adult-onset LHON patients were not included in the analysis due to unspecified ages of onset. Moreover, LHON+ patients began to experience neurological or ophthalmological disorders at a younger age than classical LHON patients did. The two conditions mentioned above both contribute to the younger median age of onset in the LHON/LHON+ group.

The m.10197G>A variant acts as a pathogenic mutation in both homoplasmic and heteroplasmic forms. The heteroplasmy level is a key determinant of pathogenicity and varies among patients [32]. A heteroplasmy level threshold of 37% in blood was reported for the clinical manifestation of LS in children across all detectable pathogenic mtDNA variants [21]. Our meta-analysis did not identify a threshold for the pathogenicity of the m.10197G>A variant. In our review, the lowest reported pathogenetic heteroplasmy of m.10197G>A in blood was 18.6% in an adult-onset LS patient [27]. The m.10197G>A mutation load among LHON/LHON+ patients varied from 10% [33] to 100%, with more than 80% of them having a homoplasmic mutation.

Heteroplasmy level variations contribute to divergent phenotypes in the mitochondrial DNA disorders [34]. Additionally, mutation levels vary over time and between tissues, further complicating phenotypic outcomes [35]. Our study investigated factors associated with different phenotypes in patients with the m.10197G>A variant, revealing that older age at onset or higher heteroplasmy levels were associated with LHON/LHON+ manifestation. In the LS/LS+ group, patients with earlier disease onset, had a greater mutation load. A similar inverse correlation between mutation load and age of onset has also been reported in patients with MELAS [36]. To our knowledge, this study is the first to explore this relationship in patients with LS, particularly in a cohort with a disease onset ranging from infancy to adulthood.

Incomplete penetrance is a characteristic of LHON, whereby some individuals with homoplasmic mutations remain asymptomatic throughout their lifetime [37]. This phenomenon was also observed in the participants carrying the m.10197G>A variant. In our review, six asymptomatic participants from four pedigrees of LS+ or LHON+ had mutation loads ranging from 37 to 100%. Potential penetrance-modifying nuclear and mitochondrial digenic mechanisms have been documented in LHON patients with the *YARS*/m.3635G>A mutation [38], which may offer insight into the phenomenon of incomplete penetrance. However, further investigation is needed to fully clarify the incomplete penetrance associated with the m.10197G>A variant.

In the absence of a specific therapeutic approach for mitochondrial disease resulting from the m.10197G>A variant, a cocktail of mitochondrial cofactors has been commonly prescribed to affected patients. The significantly greater proportion of stable or worsening outcomes observed in the LS/LS+ group is primarily due to their extensive and pronounced neurological impairment at baseline, and developmental delay and compromised daily function, exacerbated by the early age of disease onset.

This case report and review are subject to several limitations. The mother of the proband was found to carry the m.10197G>A mutation and was clinically suspected of having mitochondrial encephalopathy based on her reported medical history. However, she was excluded from the review because her phenotype could not be verified without neuroimaging and a neurological examination due to her refusal. The clinical severity of some mitochondrial diseases, such as neuropathy, ataxia, and retinitis pigmentosa (NARP)/maternally inherited LS, is increasingly associated with the level of heteroplasmic mtDNA mutations [39]. In the absence of sufficient neuroimaging data and a standardized method for assessing the severity of neuroimaging and functional impairments across the studies included in this review, an analysis of the associations among clinical severity, neuroimaging severity and mutation load was not feasible.

## Conclusion

This case report of a proband diagnosed with adult-onset LS/LDYT overlap syndrome broadens the clinical spectrum observed in adult-onset patients with the m.10197G>A mutation. A review of the literature revealed a wide range of phenotypes resulting from this mutation. Individuals carrying the m.10197G>A mutation are more likely to present as LS/LS+ or LHON/LHON+, with those with an older age of onset or a higher mutation load being more likely to present as LHON/LHON+. For Individuals who carry the m.10197G>A mutation but are asymptomatic, there is a possibility of that they may remain unaffected for the entirety of their lives. However, if these individuals develop neurological impairment before the age of 59 years, especially those with LS-like or LHON-like presentations, the etiological diagnosis should prioritize the pathogenicity of the mutation, regardless of the heteroplasmy level of the m.10197G>A variant. Patients diagnosed with LS/LS+ have an extremely high likelihood of experiencing a worse outcome.

The identification of individuals at risk of developing specific phenotypes could improve prognostic counseling for patients and their family members carrying this mutation. These findings may also provide insights into other mtDNA mutations and the development of diverse phenotypes across different ages, from infancy to adulthood.

## Supplementary Information

Additional file 1.

Additional file 2.

## Data Availability

The authors confirm that the data supporting the findings of this study are available within the article and its supplementary materials.

## References

[1] Galkin A, Moncada S. Modulation of the conformational state of mitochondrial complex I as a target for therapeutic intervention. Interface Focus. 2017;7:20160104. 28382200 10.1098/rsfs.2016.0104 PMC5311904

[2] Kirby DM, Crawford M, Cleary MA, Dahl H-HM, Dennett X, Thorburn DR. Respiratory chain complex I deficiency: an underdiagnosed energy generation disorder. Neurology. 1999;52:1255. 10214753 10.1212/wnl.52.6.1255

[3] Kirby DM, Salemi R, Sugiana C, et al. NDUFS6 mutations are a novel cause of lethal neonatal mitochondrial complex I deficiency. J Clin Investig. 2004;114:837–45. 15372108 10.1172/JCI20683 PMC516258

[4] Valente L, Piga D, Lamantea E, et al. Identification of novel mutations in five patients with mitochondrial encephalomyopathy. Biochim Biophys Acta. 2009;1787:491–501. 18977334 10.1016/j.bbabio.2008.10.001

[5] Chae JH, Lee JS, Kim KJ, et al. A novel ND3 mitochondrial DNA mutation in three Korean children with basal ganglia lesions and complex I deficiency. Pediatr Res. 2007;61:622–4. 17413873 10.1203/pdr.0b013e3180459f2d

[6] Wang K, Takahashi Y, Gao ZL, et al. Mitochondrial ND3 as the novel causative gene for Leber hereditary optic neuropathy and dystonia. Neurogenetics. 2009;10:337–45. 19458970 10.1007/s10048-009-0194-0

[7] Leng Y, Liu Y, Fang X, et al. The mitochondrial DNA 10197G>A mutation causes MELAS/Leigh overlap syndrome presenting with acute auditory agnosia. Mitochondrial DNA. 2015;26:208–12. 24708134 10.3109/19401736.2014.905860

[8] Shi Y, Chen G, Sun D, et al. Phenotypes and genotypes of mitochondrial diseases with mtDNA variations in Chinese children: a multi-center study. Mitochondrion. 2022;62:139–50. 34800692 10.1016/j.mito.2021.11.006

[9] Chang X, Wu Y, Zhou J, Meng H, Zhang W, Guo J. A meta-analysis and systematic review of Leigh syndrome: clinical manifestations, respiratory chain enzyme complex deficiency, and gene mutations. Medicine. 2020;99:e18634. 32000367 10.1097/MD.0000000000018634 PMC7004636

[10] Ardissone A, Ferrera G, Lamperti C, et al. Phenotyping mitochondrial DNA-related diseases in childhood: a cohort study of 150 patients. Eur J Neurol. 2023;30:2079–91. 37038312 10.1111/ene.15814

[11] Wong T, Belaramani KM, Chan C, et al. Mitochondrial diseases in Hong Kong: prevalence, clinical characteristics and genetic landscape. Orphanet J Rare Dis. 2023;18:43. 36859275 10.1186/s13023-023-02632-6 PMC9979401

[12] Nogueira C, Pereira C, Silva L, et al. The genetic landscape of mitochondrial diseases in the next-generation sequencing era: a Portuguese cohort study. Front Cell Dev Biol. 2024;12:1331351. 38465286 10.3389/fcell.2024.1331351 PMC10920333

[13] Severino M, Nesti C, Rubegni A, Tolomeo D, Santorelli FM. The features of the m.10197G>A mtDNA mutation. J Neurol Sci. 2019;400:184–5. 30978516 10.1016/j.jns.2019.04.005

[14] Durrleman C, Grevent D, Aubart M, et al. Clinical and radiological description of 120 pediatric stroke-like episodes. Eur J Neurol. 2023;30:2051–61. 37046408 10.1111/ene.15821

[15] Gilhooley MJ, Raoof N, Yu-Wai-Man P, Moosajee M. Inherited optic neuropathies: real-world experience in the paediatric neuro-ophthalmology clinic. Genes (Basel). 2024;15:188. 38397177 10.3390/genes15020188 PMC10888158

[16] Schaefer AM, Phoenix C, Elson JL, McFarland R, Chinnery PF, Turnbull DM. Mitochondrial disease in adults: a scale to monitor progression and treatment. Neurology. 2006;66:1932–4. 16801664 10.1212/01.wnl.0000219759.72195.41

[17] Page MJ, McKenzie JE, Bossuyt PM, et al. The PRISMA 2020 statement: an updated guideline for reporting systematic reviews. BMJ. 2021;372:n71. 33782057 10.1136/bmj.n71 PMC8005924

[18] Stewart LA, Clarke M, Rovers M, et al. Preferred reporting items for a systematic review and meta-analysis of individual participant data: the PRISMA-IPD statement. JAMA. 2015;313:1657–65. 25919529 10.1001/jama.2015.3656

[19] Bandelt HJ, Salas A, Taylor RW, Yao YG. Exaggerated status of “novel” and “pathogenic” mtDNA sequence variants due to inadequate database searches. Hum Mutat. 2009;30:191–6. 18800376 10.1002/humu.20846

[20] Herman SW. Evidence summary: google scholar could be used as a stand-alone resource for systematic reviews. Donald Barbara Zucker Sch Med. 2015;10:147–9.

[21] Stenton SL, Zou Y, Cheng H, et al. Leigh syndrome: a study of 209 patients at the Beijing children’s hospital. Ann Neurol. 2022;91:466–82. 35094435 10.1002/ana.26313

[22] Fantini M, Asanad S, Karanjia R, Sadun A. Hormone replacement therapy in Leber’s hereditary optic neuropathy: accelerated visual recovery in vivo. J Curr Ophthalmol. 2019;31:102–5. 30899856 10.1016/j.joco.2018.10.003 PMC6407313

[23] Huang TL, Wang JK, Yoong Pang C, Tsai RK. Leber’s hereditary optic neuropathy associated with the m.10197G>A mutation. J Clin Exp Ophthalmol. 2017;8:1000673.

[24] Chen Z, Zhao Z, Ye Q, et al. Mild clinical manifestation and unusual recovery upon coenzyme Q10 treatment in the first Chinese Leigh syndrome pedigree with mutation m.10197 G>A. Mol Med Rep. 2015;11:1956–62. 25384404 10.3892/mmr.2014.2911

[25] Wei Y, Cui L, Peng B. Mitochondrial DNA mutations in late-onset Leigh syndrome. J Neurol. 2018;265:2388–95. 30128709 10.1007/s00415-018-9014-5

[26] Cipriano E, Vecchio D, Mazzini L, et al. A young male with walking difficulties and subacute brainstem dysfunction: adult-onset Leigh syndrome. J Neurol Sci. 2021;429:119363.

[27] Wei Y, Qian M, Yang Y. Extended spinal cord involvement in adult-onset Leigh syndrome due to mitochondrial 10197G >A mutation. Neurol Sci. 2022;43:6997–7000. 35907985 10.1007/s10072-022-06305-3

[28] Wei Y, Huang Y, Yang Y, Qian M. MELAS/LS overlap syndrome associated with mitochondrial DNA mutations: clinical, genetic, and radiological studies. Front Neurol. 2021;12:648740. 34025555 10.3389/fneur.2021.648740 PMC8137909

[29] Blickhäuser B, Stenton SL, Neuhofer CM, et al. Digenic Leigh syndrome on the background of the m.11778G>A Leber hereditary optic neuropathy variant. Brain. 2024;147:1967–74. 38478578 10.1093/brain/awae057 PMC11146415

[30] Neuhofer CM, Prokisch H. Digenic inheritance in rare disorders and mitochondrial disease—crossing the frontier to a more comprehensive understanding of etiology. Int J Mol Sci. 2024;25:4602. 38731822 10.3390/ijms25094602 PMC11083678

[31] Yu-Wai-Man P, Newman NJ, Carelli V, et al. Natural history of patients with Leber hereditary optic neuropathy—results from the REALITY study. Eye. 2022;36:818–26. 33911213 10.1038/s41433-021-01535-9 PMC8956580

[32] Schon KR, Ratnaike T, van den Ameele J, Horvath R, Chinnery PF. Mitochondrial diseases: a diagnostic revolution. Trends Genet. 2020;36:702–17. 32674947 10.1016/j.tig.2020.06.009

[33] Solyman O, MacIntosh P. Leber hereditary optic neuropathy in a mother and daughter associated with m.10197G>A mutation. J Neuro-Ophthal. 2019;39:142. 10.1097/WNO.0000000000000714 30199507

[34] Rossignol R, Faustin B, Rocher C, Malgat M, Mazat J-P, Letellier T. Mitochondrial threshold effects. Biochem J. 2003;370:751–62. 12467494 10.1042/BJ20021594 PMC1223225

[35] Magrinelli F, Balint B, Bhatia KP. Challenges in clinicogenetic correlations: one gene—many phenotypes. Mov Disord Clin Pract. 2021;8:299–310. 33816657 10.1002/mdc3.13165 PMC8015894

[36] Chae HW, Na JH, Kim HS, Lee YM. Mitochondrial diabetes and mitochondrial DNA mutation load in MELAS syndrome. Eur J Endocrinol. 2020;183:505–12. 33107434 10.1530/EJE-20-0189

[37] La Morgia C, Maresca A, Caporali L, Valentino ML, Carelli V. Mitochondrial diseases in adults. J Intern Med. 2020;287:592–608. 32463135 10.1111/joim.13064

[38] Jin X, Zhang J, Yi Q, et al. Leber’s hereditary optic neuropathy arising from the synergy between ND1 3635G>A mutation and mitochondrial YARS2 mutations. Investig Opthalmol Vis Sci. 2021;62:22. 10.1167/iovs.62.7.22 PMC8237128 34156427

[39] Carelli V, Baracca A, Barogi S, et al. Biochemical-clinical correlation in patients with different loads of the mitochondrial DNA T8993G mutation. Arch Neurol. 2002;59:264–70. 11843698 10.1001/archneur.59.2.264

