# Supplementary figures and images for: Clinical spectrum, treatment and outcomes of the m.10197G>A mutation in MT-ND3: a case report, systematic review and meta-analysis
Source: Orphanet J Rare Dis. 2025 Feb 8;20:59. doi: 10.1186/s13023-025-03588-5 (PMC11806901; doi:10.1186/s13023-025-03588-5)

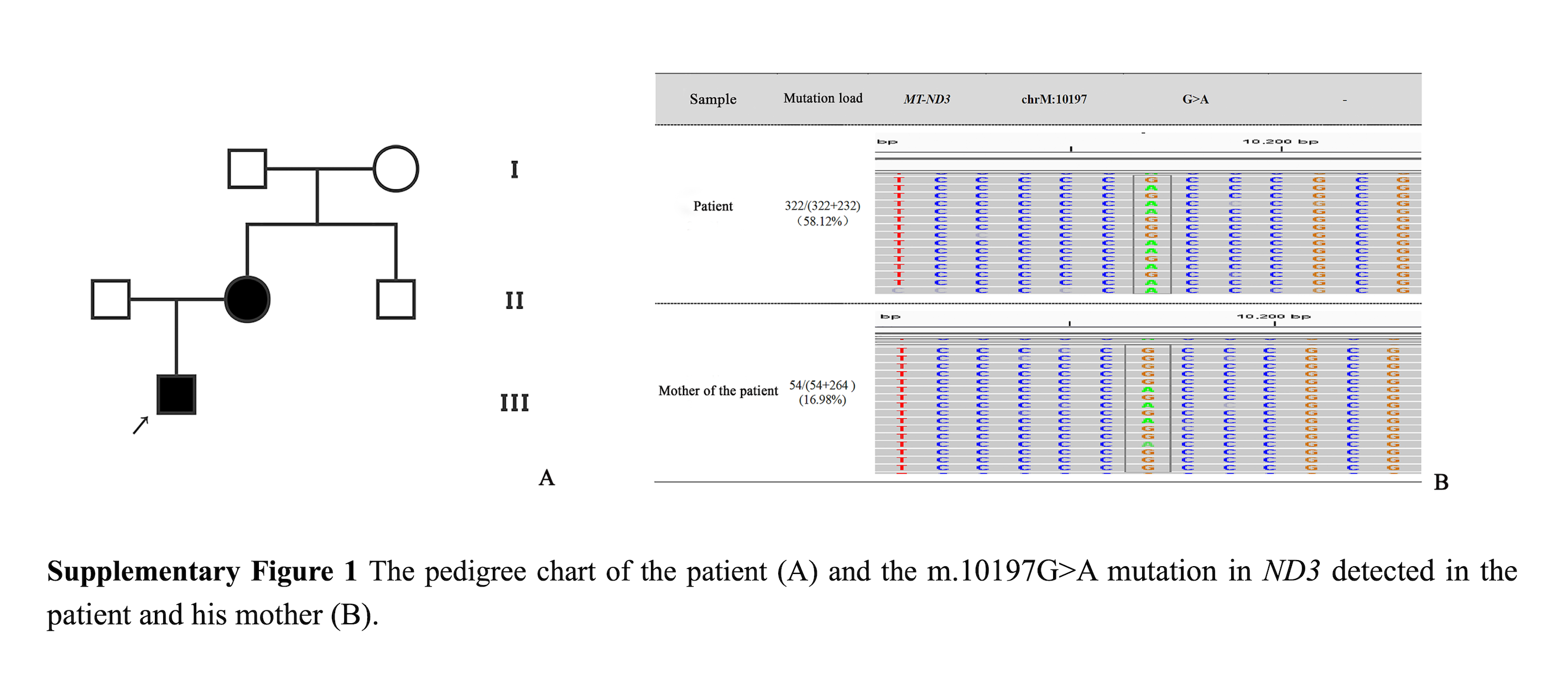

Supplement: Supplementary file 1 — Additional file 1. [file 13023_2025_3588_MOESM1_ESM.tiff]
