# Supplementary material for: Clinical spectrum, treatment and outcomes of the m.10197G>A mutation in MT-ND3: a case report, systematic review and meta-analysis
Source: Orphanet J Rare Dis. 2025 Feb 8;20:59. doi: 10.1186/s13023-025-03588-5 (PMC11806901; doi:10.1186/s13023-025-03588-5)
Supplement: Supplementary file 2 — Additional file 2. [file 13023_2025_3588_MOESM2_ESM.pdf]

Supplementary Table 1 Patients with m.10197G>A mutation reported in the present study and previous literature.

No. of article	References	Country / District	No. of carrier	Gender / presenting age (year)	Onset age (year)	Family History	Mutation Load % (sample)	Phenotype Spectrum	Treatment	Outcome
1	Present study	China	P1	M / Early 20s	Young adult	Yes	58.1% (Leu)<br>77.1% (Mus)	Adult-onset LS / LDYT overlap syndrome	Idebenone, ubidecarenone	Stable at 1-year follow-up
2	Gilhooley, el al., 2024[1]	England	P2	NA / Child, NA	Child, NA	NA	Homoplasmic (NA)	LHON	NA	NA
3	Baldo, et al., 2023[2]	Portugal	P3	M / 7	Child, NA	NA	Homoplasmic (Mus)	LS and epilepsy	NA	NA
4	Nogueira, et al., 2023[3]	Portugal	P4	M / 4	Child, NA	NA	Homoplasmic (Mus)	LS	NA	NA
5	Durrleman, et al., 2023[4]	France	P5	NA / Child, NA	Child, NA	NA	NA	MELAS	NA	NA
6	Atdissone, et al., 2023[5]	Italy	P6	NA / Child, NA	Child, NA	NA	99.6% (Leu), 98% (Mus)	LS	Mitochondrial cofactor “cocktail”	NA
			P7	NA / Child, NA		NA		LS		NA
			P8	NA / Child, NA		NA		LS		NA
			P9	NA / Child, NA		NA	99-100% (Leu), 95-100% (Mus)	LS		NA
			P10	NA / Child, NA		NA		LS		NA
7	Stenton, et al., 2022[6]	China	P11	NA / NA	0	NA	Homoplasmic (Leu)	LS	NA	NA
			P12	NA / NA	5.75	NA	83% (Leu)	Late-onset LS	NA	NA
			P13	NA / NA	0.42	NA	Homoplasmic (Leu)	LS	NA	NA
			P14	NA / NA	4.93	NA	97% (Leu)	Late-onset LS	NA	NA
			P15	NA / NA	9.5	NA	98% (Leu)	Late-onset LS	NA	NA
			P16	NA / NA	2.25	NA	89% (Leu)	Late-onset LS	NA	NA
			P17	NA / NA	0.25	NA	99% (Leu)	LS	NA	NA
8	Wei, et al., 2022[7]	China		*Family*		Yes				
			P18	M / 19	14		68% (Leu), 93% (Uri)	Late-onset LS	NA	NA

			P19	F / 49	22		18.6% (Leu), 61% (Uri)	Adult-onset LS	NA	NA
9	Wu, et al., 2022[8]	China	P20	NA / Child, NA	Child, NA	None	99.6% (Leu)	LS	NA	NA
10	Zhao, et al., 2022[9]	China	P21	NA / Adult, NA	Adult, NA	NA	NA	LHON	NA	NA
11	Hechmi, et al., 2022[10]	Tunisia	P22	M / 5	1	Yes	87.7% (Leu)	LS	NA	NA
12	Cipriano, et al., 2021[11]	Italy	P23	M / 36	25	None	NA	Adult-onset LS	acetylcarnitine, ubidecarenone, tracheostomy with not-invasive ventilation	alive at 7 months after diagnosis
13	Wei, et al., 2021[12]	China	P24	M / 20	14	None	61.5% (Leu)	MELAS/LS overlap syndrome	NA	NA
14	Gramegna, et al., 2021[13]	Italy	P25	F / 36	12	NA	85.0% (Leu), 98.0% (Uri)	MELAS	NA	NA
15	Cui, et al., 2020[14]	China	P26	M / 19	19	None	Heteroplasmy (Leu)	LHON	NA	No obvious visual improvement at the 1-year
16	Chan, et al., 2020[15]	USA	P27	F / 20	20	NA	51% (NA)	LHON	Idebenone, vitamin C	Mild visual improvement
17	Tolomeo, et al., 2019[16]	Italy	P28	M / 7	6	None	Homoplasmic (Leu, Uri, Mus, Fib)	Dystonia and LS	NA	NA
			P29	M / 5	0.75	None	99.9% (Leu), 99.1% (Mus), 99.4% (Fib)	Dystonia and LS	NA	NA
			P30	M / 4	1.33	None	96.4% (Mus), 95.2% (Fib)	LS and arterial malformations	NA	NA

18	Solyman, et al., 2019[17]	USA	P31	F / 48	48	Yes	10-30% (NA)	LHON	Idebenone	Visual acuity improved at 4-month follow-up
19	Fantini, et al., 2019[18]	USA		*Family*		Yes				
			P32	F / 48	48		NA	LHON	Idebenone, vitamin C, hormone replacement therapy	Complete reversal of vision loss by eight months post-therapy
			P33	F / NA	58		NA	LHON	NA	Vision loss
20	Pereira, et al., 2019[19]	Brazil	P34	M / 1.41	0.91	None	Homoplasmic (Mus)	LS	NA	Died 20 days later 0(3 years and 2 months of age)
21	Wei, et al., 2018[20]	China	P35	F / NA	2	None	92% (Leu)	Late-onset LS	Coenzyme Q10, vitamins C, E, B1, and B2.1	NA
			P36	M / NA	6	None	83% (Leu)	Late-onset LS	Coenzyme Q10, vitamins C, E, B1, and B2.	NA
			P37	M / NA	14	None	68% (Leu)	Late-onset LS	Coenzyme Q10, vitamins C, E, B1, and B2.	NA
			P38	M / NA	22	None	45% (Leu)	Adult-onset LS	Coenzyme Q10, vitamins C, E, B1, and B2.	NA
22	Huang, et al., 2017[21]	Taiwan	P39	M / 23	23	None	64.4% (Leu)	LHON	Idebenone, vitamin C	Visual recovery at 1-year follow-up
23	Zhang, et al., 2018[22]	China	P40	NA / NA	NA	NA	NA	MELAS	NA	NA
24	Lee, et al., 2016[23]	Korea	P41	F / 6	1.33	NA	NA	LS	NA	Stable
			P42	M / 16	2.25	NA	NA	Late-onset LS	NA	Deterioration
			P43	F / 13	3	NA	NA	Late-onset LS	NA	Stable

25	Leng, et al., 2015[24]	China	P44	M / 14	14	None	61.1% (Leu), 75.8% (Uri), 85.1% (Mus)	MELAS/LS overlap syndrome	Vitamin B and several coenzymes	Stable
26	Chen, et al., 2015[25]	China		*Family*		Yes				
			P45	F / 16	15		95.0% (Leu), 97.0% (Mus)	Late-onset LS	Coenzyme Q10	Improved at 3-month follow-up
			HC1	F / NA (mother)			95% (Leu)	Asymptomatic carrier	/	/
			HC2	M / NA (brother)			39% (Leu)	Asymptomatic carrier	/	/
27	Calvo, et al., 2010[26]	Australia	P46	NA / NA	3	None	90% (Leu)	Mitochondrial encephalopathy	NA	NA
28	Wang, et al., 2009[27]	China		*Family*		Yes				
			P47	M / NA	5		Homoplasmic (Leu)	LDYT	NA	NA
			P48	M / NA	8		Homoplasmic (Leu)	LDYT	NA	NA
			P49	F / NA	8		Homoplasmic (Leu)	LDYT	NA	NA
			P50	F / NA	5		Homoplasmic (Leu)	LDYT	NA	NA
			P51	F / 20	8		Homoplasmic (Leu)	LDYT	NA	NA
			P52	M / 18	5		Homoplasmic (Leu)	LDYT	NA	NA
			P53-62	5M / NA and 5F / NA	14-30		Homoplasmic (Leu)	LHON	NA	NA
			HC3-HC4	1M / NA and 1F / NA	/		Homoplasmic (Leu)	Asymptomatic carrier	/	/
29	Naess, et al., 2009[28]	Sweden	P63	M / NA	7	NA	50% (Mus), 31% (Fib)	Late-onset LS	NA	NA
30	Valente, et al., 2009[29]	Italy	P64	NA / NA	0.58	NA	NA	LS	NA	NA
31	Chae, et al., 2007[30]	Korean	P65	F / 9	7	Yes	98% (Mus)	Late-onset LS	NA	NA
			P66	M / NA	4	Yes	86% (Mus)	Late-onset LS	NA	NA
			P67	M / NA	0.25	None	80% (Mus)	LS	NA	NA
32	Sarzi, et al., 2007[31]	France		*Family 1*		Yes				
			P68	F / 41 (mother)	14		67% (Leu)	Dystonia	NA	Stable

			P69	M / NA	0.08		Homoplasmic (Mus)	Dystonia and LS	NA	Died at 5 mon
			P70	M / NA	0.16		Homoplasmic (Leu, liv)	Dystonia and LS	NA	Died at 2 mon
			P71	F / NA	0.16		Homoplasmic (Mus)	Dystonia	NA	Died at 8 mon
				Family 2		Yes				
			HC5	F / 34 (mother)	/		50% (Leu)	Asymptomatic carrier	/	/
			P72	M / NA	0.33		/	Dystonia and LS, epilepsy	NA	Died at 11-mon
			P73	M / 5	0.41		Homoplasmic (Mus, Fib)	Dystonia and LS, epilepsy	NA	Developmental delay and deterioration
				Family 3		Yes				
			P74	F / 37 (mother)	6		74% (Leu), 96% (Mus)	Dystonia and late-onset LS	NA	Stable
			HC6	F / 7	/		73% (Leu)	Asymptomatic carrier	/	/
			P75	F / 5	0.41		Homoplasmic (Leu)	Seizure and development delay	NA	Seizure and developmental delay
			P76	M / NA	2		/	Encephalopathy and seizure	NA	Died at 2-year
			P77	M / NA	0.41		Homoplasmic (Leu, Mus, Cybrids)	Dystonia and LS, seizure	NA	Died at 1-year
**33**	Tchikviladzé, et al. 2007[32]	France	P78	M / 30	6	NA	<50% (Leu), 100% (Mus), 70% (buccal mucosa cells)	Late-onset LS	NA	Deterioration
**34**	Kirby, et al., 2004[33]	Australia	P79	F / NA	2	Yes	Homoplasmic (Mus)	LS-like	NA	Alive at 13 years

F, Female. LDTY, Leber hereditary optic neuropathy and dystonia. HC, healthy carrier. Leu, leukocytes. LHON, Leber hereditary optic neuropathy. Liv, liver. LS, Leigh syndrome. M, male. MELAS, mitochondrial encephalopathy

with lactate acidosis and stroke-like episodes. Mon, months. Mus, muscle. NA, not available. Uri, urine sediment. P, patient.

References

1. Gilhooley MJ, Raoof N, Yu-Wai-Man P, Moosajee M. Inherited optic neuropathies: real-world experience in the paediatric neuro-ophthalmology clinic. Genes. 2024;15:188.

2. Baldo MS, Nogueira C, Pereira C, Janeiro P, Ferreira S, Lourenço CM, et al. Leigh syndrome spectrum: a portuguese population cohort in an evolutionary genetic era. Genes. 2023;14:1536.

3. Nogueira C, Pereira C, Silva L, Laranjeira M, Lopes A, Neiva R, et al. The genetic landscape of mitochondrial diseases in the next-generation sequencing era: a Portuguese cohort study. Front Cell Dev Biol. 2024;12:1331351.

4. Durrleman C, Grevent D, Aubart M, Kossorotoff M, Roux CJ, Kaminska A, et al. Clinical and radiological description of 120 pediatric stroke-like episodes. Eur J Neurol. 2023;30:2051–61.

5. Ardissone A, Ferrera G, Lamperti C, Tiranti V, Ghezzi D, Moroni I, et al. Phenotyping mitochondrial DNA-related diseases in childhood: A cohort study of 150 patients. Eur J Neurol. 2023;30:2079–91.

6. Stenton SL, Zou Y, Cheng H, Liu Z, Wang J, Shen D, et al. Leigh syndrome: a study of 209 patients at the beijing children's hospital. Ann Neurol. 2022;91:466–82.

7. Wei Y, Qian M, Yang Y. Extended spinal cord involvement in adult-onset Leigh syndrome due to mitochondrial 10197G>A mutation. Neurol Sci. 2022;43:6997–7000.

8. Wu T, He F, Xiao N, Han Y, Yang L, Peng J. Phenotype-genotype analysis based on molecular classification in 135 children with mitochondrial disease. Pediatr Neurol. 2022;132:11–8.

9. Zhao J, Zhang Q, Wang J. Magnetic resonance imaging findings in the pregeniculate visual pathway in leber hereditary optic neuropathy. J Neuro-Ophthalmol. 2022;42:E153–8.

10. Hechmi M, Charif M, Kraoua I, Fassatoui M, Dallali H, Desquiret-Dumas V, et al. Next generation sequencing of Tunisian Leigh syndrome patients reveals novel variations: impact for diagnosis and treatment. Biosci Rep. 2022;BSR20220194.

11. Cipriano E, Vecchio D, Mazzini L, Strigaro G, Cantello R, Comi G Pietro, et al. A young male with walking difficulties and subacute brainstem dysfunction: Adult-onset Leigh syndrome. J Neurol Sci. 2021;429:119363.

12. Wei Y, Huang Y, Yang Y, Qian M. MELAS/LS Overlap syndrome associated with mitochondrial DNA mutations: clinical, genetic, and radiological studies. Front Neurol. 2021;12:648740.

13. Gramegna LL, Evangelisti S, di Vito L, la Morgia C, Maresca A, Caporali L, et al. Brain MRS correlates with mitochondrial dysfunction biomarkers in MELAS-associated mtDNA mutations.

Ann Clin Transl Neurol. 2021;8:1200–11.

14. Cui S, Yang L, Jiang H, Peng J, Shang J, Wang J, et al. Clinical features of chinese sporadic leber hereditary optic neuropathy caused by rare primary mtDNA Mutations. J Neuro-Ophthalmol. 2020;40:30–6.

15. Chan J, Sadun A. Leber's Hereditary Optic Neuropathy-like syndrome in heteroplasmic mitochondrial mutations. Acta Ophthalmol. 2021;99:1755-3768.

16. Tolomeo D, Rubegni A, Severino M, Pochiero F, Bruno C, Cassandrini D, et al. Clinical and neuroimaging features of the m.10197G>A mtDNA mutation: New case reports and expansion of the phenotype variability. J Neurol Sci. 2019;399:69–75.

17. Solyman O, MacIntosh P. Leber hereditary optic neuropathy in a mother and daughter associated with m.10197G>A mutation. J Neuro-Ophthalmol. 2019;39:142–142.

18. Fantini M, Asanad S, Karanjia R, Sadun A. Hormone replacement therapy in Leber's hereditary optic neuropathy: accelerated visual recovery in vivo. J Curr Ophthalmol. 2019;31:102–5.

19. Pereira C, Souza CF de, Vedolin L, Vairo F, Lorea C, Sobreira C, et al. Leigh syndrome due to mtDNA pathogenic variants. J Inborn Errors Metab Screen. 2019;7:e20180003.

20. Wei Y, Cui L, Peng B. Mitochondrial DNA mutations in late-onset Leigh syndrome. J Neurol. 2018;265:2388–95.

21. Huang TL, Wang JK, Yoong Pang C, Kung Tsai R. Leber's hereditary optic neuropathy associated with the m.10197G>A mutation. J Clin Exp Ophthalmol. 2017;8:1000673.

22. Zhang Z, Zhao D, Zhang X, Xiong H, Bao X, Yuan Y, et al. Survival analysis of a cohort of Chinese patients with mitochondrial encephalomyopathy with lactic acidosis and stroke-like episodes (MELAS) based on clinical features. J Neurol Sci. 2018;385:151–5.

23. Lee JS, Kim H, Lim BC, Hwang H, Choi J, Kim KJ, et al. Leigh syndrome in childhood: neurologic progression and functional outcome. J Clin Neurol. 2016;12:181.

24. Leng Y, Liu Y, Fang X, Li Y, Yu L, Yuan Y, et al. The mitochondrial DNA 10197 G > A mutation causes MELAS/Leigh overlap syndrome presenting with acute auditory agnosia. Mitochondrial DNA. 2015;26:208–12.

25. Chen Z, Zhao Z, Ye Q, Chen Y, Pan X, Sun B, et al. Mild clinical manifestation and unusual recovery upon coenzyme Q10 treatment in the first Chinese Leigh syndrome pedigree with mutation m.10197 G>A. Mol Med Rep. 2015;11:1956–62.

26. Calvo SE, Tucker EJ, Compton AG, Kirby DM, Crawford G, Burtt NP, et al. High-throughput, pooled sequencing identifies mutations in NUBPL and FOXRED1 in human complex I deficiency. Nat Genet. 2010;42:851–8.

27. Wang K, Takahashi Y, Gao ZL, Wang GX, Chen XW, Goto J, et al. Mitochondrial ND3 as the novel causative gene for Leber hereditary optic neuropathy and dystonia. Neurogenetics. 2009;10:337–45.

28. Naess K, Freyer C, Bruhn H, Wibom R, Malm G, Nennesmo I, et al. mtDNA mutations are a common cause of severe disease phenotypes in children with Leigh syndrome. BBA-Bioenergetics. 2009;1787:484–90.

29. Valente L, Piga D, Lamantea E, Carrara F, Uziel G, Cudia P, et al. Identification of novel mutations in five patients with mitochondrial encephalomyopathy. Biochim Biophys Acta. 2009;1787:491–501.

30. Chae JH, Lee JS, Kim KJ, Hwang YS, Bonilla E, Tanji K, et al. A novel ND3 mitochondrial dna mutation in three korean children with basal ganglia lesions and complex i deficiency. Pediatr Res. 2007;61:622–4.

31. Sarzi E, Brown MD, Lebon S, Chretien D, Munnich A, Rotig A, et al. A novel recurrent mitochondrial DNA mutation in ND3 gene is associated with isolated complex I deficiency causing leigh syndrome and dystonia. Am J Med Genet A. 2007;143A:33–41.

32. Tchikviladzé M, Laforêt P, Eymard B, Delbos F, Filaut S, Lombès A, et al. A novel mutation in the mitochondrial ND3 gene causing Leigh syndrome with late-onset neurological decline. Neuromuscular Disord. 2007;17:769.

33. Kirby DM, Salemi R, Sugiana C, Ohtake A, Parry L, Bell KM, et al. NDUFS6 mutations are a novel cause of lethal neonatal mitochondrial complex I deficiency. J Clin Invest. 2004;114:837–45.
